# Efficacy and Safety of Different Maintenance Doses of Caffeine Citrate for Treatment of Apnea in Premature Infants: A Systematic Review and Meta-Analysis

**DOI:** 10.1155/2018/9061234

**Published:** 2018-12-24

**Authors:** Jing Chen, Lu Jin, Xiao Chen

**Affiliations:** ^1^Department of Neonatology, The First People's Hospital of Neijiang, Sichuan 641000, China; ^2^Department of Orthopedic Surgery, The First People's Hospital of Neijiang, Sichuan 641000, China

## Abstract

**Background:**

Caffeine is widely used for the treatment of neonatal apnea, but there is no agreement on the optimum maintenance dose for preterm infants.

**Objective:**

The aims of this meta-analysis were to compare the efficacy and safety of high versus low maintenance doses of caffeine citrate for the treatment of apnea in premature infants.

**Methods:**

Literature searches were conducted using PubMed, Cochrane Library, OVID, Embase, Web of Science, Chinese Biomedical Literature, Weipu Journal, Wanfang, and CNKI databases up to September 2018. Only randomized controlled trials (RCTs) of caffeine citrate for apnea treatment in premature infants were included. Trials were divided into those testing high maintenance doses (10−20 mg/kg daily) and low maintenance doses (5−10 mg/kg daily) for comparison. Data collection and extraction, quality assessment, and data analyses were performed according to the Cochrane standards.

**Results:**

Among the 345 studies initially identified, thirteen RCTs involving 1515 patients were included. Compared to the low-dose group, the high-dose group exhibited greater effective treatment rate (RR: 1.37, 95%CI: 1.18 to 1.60, P<0.0001) and success rate for ventilator removal (RR: 1.74, 95%CI: 1.04 to 2.90, P=0.03), but higher incidence of tachycardia (RR: 2.02, 5%CI: 1.30 to 3.12, P=0.002). The high-dose group also demonstrated lower extubation failure rate (RR: 0.5, 95%CI: 0.35 to 0.71, P=0.0001), frequency of apnea (WMD: -1.55, 95%CI: -2.72 to -0.39, P=0.009), apnea duration (WMD: -4.85, 95%CI: -8.29 to -1.40, P=0.006), and incidence of bronchopulmonary dysplasia (RR: 0.79, 95%CI: 0.68 to 0.91, P=0.002). There were no significant group differences in other adverse events including in-hospital death (P>0.05).

**Conclusions:**

Higher maintenance doses of caffeine citrate appear more effective and safer than low maintenance doses for treatment of premature apnea, despite a higher incidence of tachycardia.

## 1. Introduction

Apnea is a common condition in premature infants due to the immaturity of respiratory control mechanisms [1]. Indeed, incidence increases with younger gestational age and lower birth weight, afflicting 25% of infants under 2500 g and 80% under 1000 g [2]. Recurrent apnea can lead to respiratory failure, pulmonary hemorrhage, abnormal heart and lung function, intracranial hemorrhage, abnormal nervous system development, and even sudden death [3, 4]. Therefore, the rate of disability and mortality of infants could be significantly reduced by early and effective clinical intervention [5, 6].

At present, respiratory support and methylxanthine drugs such as theophylline, aminophylline, and caffeine are the main treatments for apnea of prematurity (AOP) [7, 8]. Caffeine has many potential advantages. It has a higher therapeutic ratio and fewer adverse reactions, is absorbed more reliably when administered enterally, and has a longer half-life than other methylxanthines. It is also effective in apneic infants unresponsive to theophylline [9, 10]. Thus, caffeine citrate has been used for the treatment of AOP in developed countries since the 1970s [11]. It was introduced in China in 2013 and has gradually replaced aminophylline as the preferred drug for AOP [12].

Although the curative efficacy of caffeine citrate for apnea treatment has been confirmed by several studies [13, 14], there is still substantial variation in the selection of maintenance dose for apnea treatment in premature infants due to the physiological particularities of this population, particularly hepatic and renal insufficiency and physical underdevelopment. Moreover, due to imperfect design and small sample size, previous studies on this issue are not convincing and no meta-analysis has been conducted on the safety and efficacy of different caffeine citrate maintenance doses for AOP. Therefore, the aims of this meta-analysis are to evaluate the efficacy and safety of low and high caffeine citrate maintenance doses for AOP treatment of premature infants by pooled analysis of existing clinical studies.

## 2. Methods

### 2.1. Data Sources and Searches

We searched for all relevant studies in the PubMed, Cochrane Library, OVID, Embase, Web of Science, Chinese Biomedical Literature, Weipu Journal, Wanfang, and CNKI databases published from inception to September 2018. The search strategy combined two areas as MeSH terms, keywords, and text words using Boolean operators: (i) “infant, premature [MeSH]” OR “infants, premature” OR “premature infant” OR “premature infants” OR “preterm infants” OR “preterm infant” OR “infant, preterm” OR “infants, preterm” OR “neonatal prematurity” OR “prematurity, neonatal”; AND (ii) “apnea [MeSH]” AND (iii) “caffeine [MeSH]” OR “1,3,7-Trimethylxanthine” OR “Vivarin” “caffedrine” OR “coffeinum” AND “dose”. Google Scholar was also searched to identify potentially relevant literature. In addition, the reference lists of included studies and all related review articles were checked for additional trials, published or unpublished. The search was limited to randomized controlled trials (RCTs) published in English or Chinese.

### 2.2. Study Selection

Studies were selected based on the following inclusion criteria. (i) Patients: participants were medically diagnosed with AOP and (first case) were younger than 37 weeks' gestational age at birth with typical apnea episode duration longer than 20 seconds or (second case) demonstrated typical apnea episodes shorter than 20 seconds but with heart rate < 100 beats/minute (bpm) or blue skin, hypoxemia, and hypotonia [15]. (ii) Interventions: all patients were given a load dose (no limits), then changed to a maintenance dose of caffeine citrate after 24 hours administered once daily by intravenous infusion and stopped 7 days after mitigation of apnea. The high dose group (HD group) received a maintenance dose of 10−20 mg/kg/day while the LD group was treated with a maintenance dose of 5−10 mg/kg/day. (iii) Outcomes: (1) primary outcomes were (a) rate of effective treatment, defined as successful evacuation within 72 hours after treatment onset, fewer than 3 apnea episodes per day, and no significant abnormalities in respiratory rhythm, (b) adverse effects such as tachycardia, electrolyte disturbance, hypertension, hyperglycemia, feed intolerance, and restlessness, and (c) in-hospital mortality. (2) Secondary outcomes were success rate of ventilator removal, extubation failure rate, frequency of apnea, apnea duration, and complications (bronchopulmonary dysplasia [BPD], retinopathy of prematurity [ROP], necrotizing enterocolitis [NEC], intraventricular hemorrhage [IVH], and periventricular leukomalacia [PVL]). (iv) Study design: only prospective RCTs were considered.

Exclusion criteria were as follows: (1) retrospective studies, cohort studies, single-case reports, animal studies, reviews, meta-analyses, posters, or abstracts; (2) study objective or intervention measures failed to meet the inclusion criteria; (3) duplicate or multiple publications of the same study; (4) studies without usable data.

### 2.3. Data Extraction and Quality Assessment

The abstracts of retrieved studies were independently reviewed by two authors (Jing Chen and Lu Jin) and full articles were examined when necessary. The data were extracted independently by these two authors and any disagreements were resolved by discussion with at least one more author (Xiao Chen) until a consensus was reached. If more than one article was published from the same cohort, the study with the most comprehensive data was selected for inclusion.

The following data was extracted: general information (first author, country of origin, publication date, number of total cases, number of males and females, mean ages, interventions, and follow-ups) and outcomes (as defined above).

Risk of bias for each study was assessed using the Cochrane Risk of Bias Tool. Bias was assessed as a judgment (high, low, or unclear) for seven domains: random sequence generation, allocation concealment, blinding of participants and personnel, blinding of outcome assessment, incomplete outcome data, selective reporting, and other sources of bias.

### 2.4. Data Synthesis and Analysis

The data were pooled using Review Manager 5.3 software. Risk ratios (RRs) were calculated for dichotomous variables in each study. Weighted mean difference (WMD) was calculated for continuous variables, and 95% confidence intervals (CIs) were determined for all effect sizes. Heterogeneity of the included studies was evaluated using Higgins I^2^. A random-effect model was used when apparent heterogeneity was detected (I^2^ ≥ 50%, P<0.05). Otherwise, a fixed effect model was used (I^2^ < 50%, P≥0.05). Potential publication bias was judged by Begg's or Egger's tests. Sensitivity analysis was performed to determine the robustness of the combined data. A p-value < 0.05 was regarded as statistically significant.

## 3. Results

**Figure 1:**
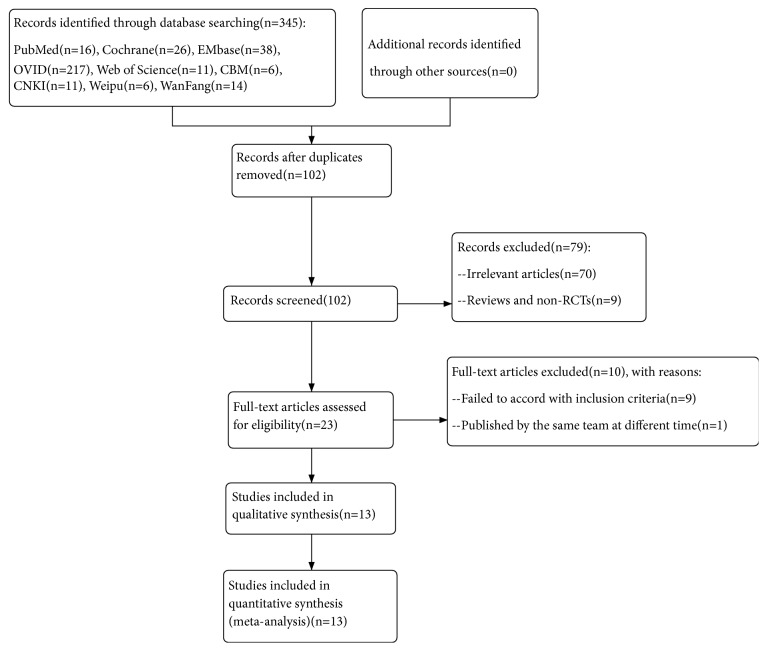
The flow chart of literature selection.

The initial literature search identified 345 citations. After removal of duplicates, 102 studies were screened for eligibility. Of these, 79 were excluded based on title and abstract review, leaving 23 full-text articles for full-text evaluation. An additional 10 studies that failed to meet the inclusion criteria were excluded, leaving 13 RTCs [16–28] for inclusion (Figure 1).

**Figure 2:**
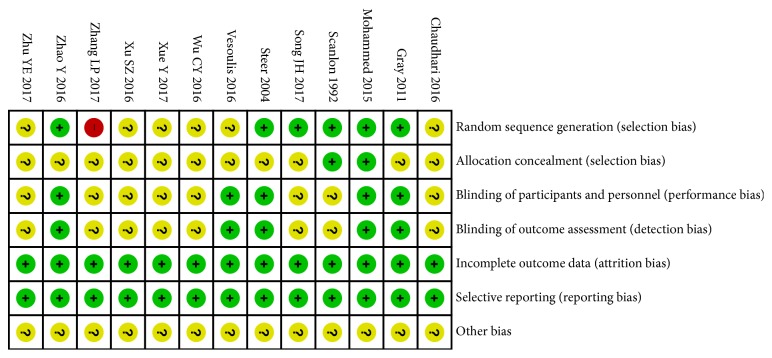
Risk of bias assessment summary of this meta-analysis.

The main characteristics of these studies are summarized in Table 1. Five studies were written in English and the other eight in Chinese. According to the Cochrane Collaboration Risk of Bias Tool, the quality of all RCTs was acceptable as all reported the method of randomization (Figure 2). Six RCTs were conducted using computer-generated lists, two used sealed envelopes, and 5 reported blinding of the doctors and participants. No trial showed an unclear bias due to incomplete outcome data or selective outcome reporting.

### 3.1. Meta-Analysis on Efficacy of Intervention

#### 3.1.1. The Effective Rate

**Figure 3:**
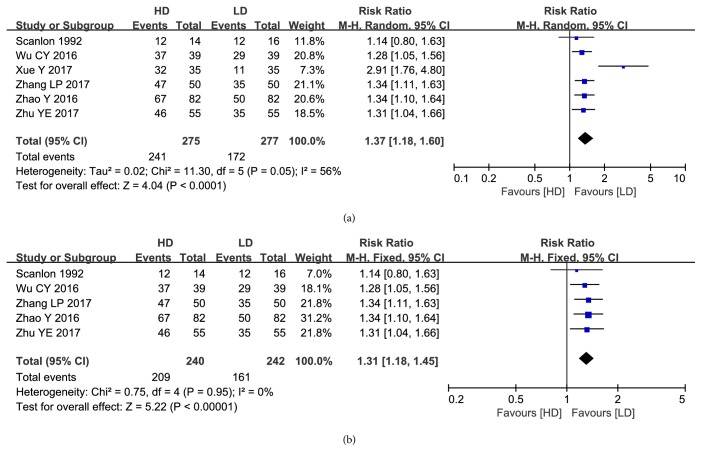
(a) Forest plot of the effective rate between HD group and LD group. (b) Forest plot of the effective rate after excluding the outlier study between HD group and LD group.

Six articles with a total of 413 infants reported the relevant data regarding efficacy rate of caffeine treatment. Data pooling revealed a significantly higher effective rate in the HD group compared to the LD group (RR: 1.37, 95%CI: 1.18 to 1.60, P<0.0001; Figure 3(a)). Sensitive analysis after excluding the outlier study also revealed a significant difference between HD group and LD group for the remaining studies with low statistical heterogeneity (RR: 1.31, 95%CI: 1.18 to 1.45, P<0.00001, I^2^=0%; Figure 3(b)).

#### 3.1.2. Adverse Effects

**Figure 4:**
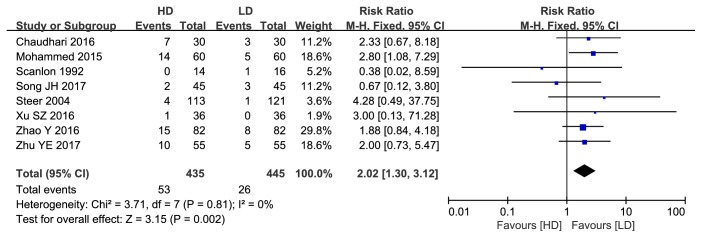
Forest plot of tachycardia between HD group and LD group.

**Figure 5:**
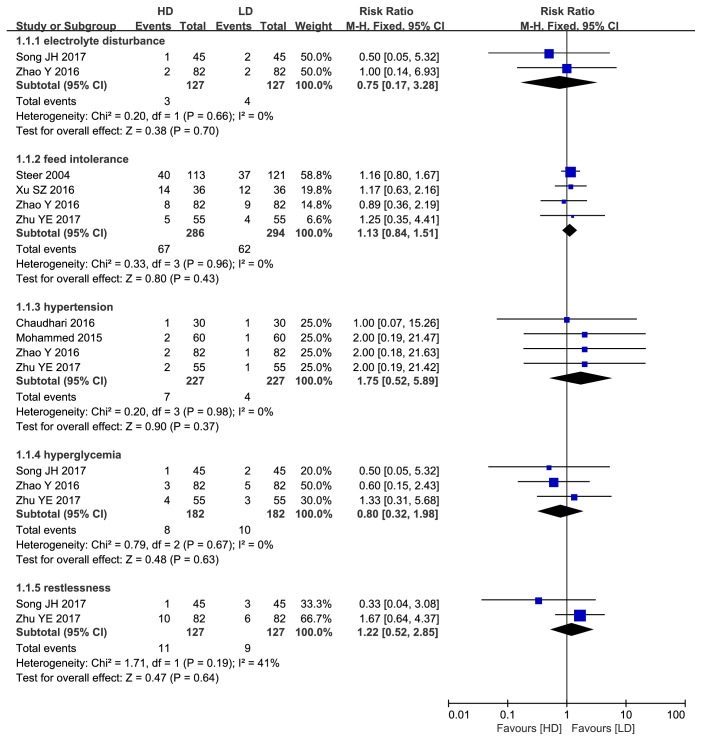
Forest plot of other adverse effects between HD group and LD group.

A total of 8 studies reported the incidence of tachycardia (435 infants in the HD group and 445 in the LD group). The incidence of tachycardia was significantly higher in the HD group than the LD group (RR: 2.02, 95%CI: 1.30 to 3.12, P=0.002; Figure 4). In contrast, there were no statistically significant differences in other adverse effects such as electrolyte disturbance, hypertension, hyperglycemia, feeding intolerance, and restlessness (P>0.05; Figure 5).

#### 3.1.3. Hospital Mortality

**Figure 6:**
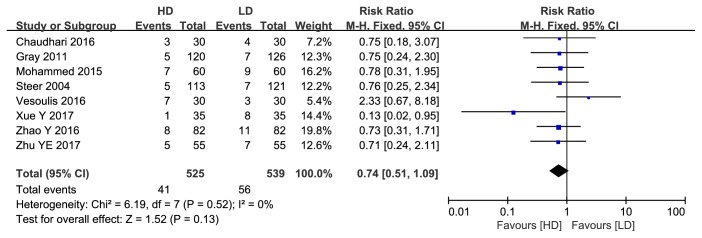
Forest plot of hospital mortality between HD group and LD group.

Data on hospital mortality were available in eight articles including 1064 infants. Data pooling revealed no significant difference between groups (RR: 0.74, 95%CI: 0.51 to 1.09, P=0.13; Figure 6).

#### 3.1.4. The Success Rate of Removal of Ventilator and Extubation Failure Rate

**Figure 7:**
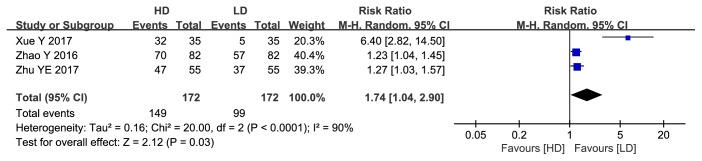
Forest plot of the success rate of ventilator removal between HD group and LD group.

**Figure 8:**
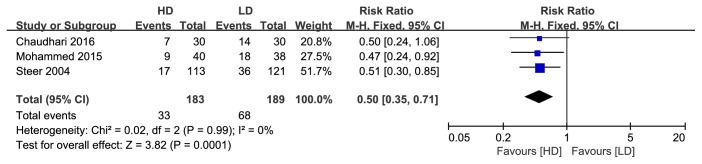
Forest plot of the extubation failure rate between HD group and LD group.

Three studies reported success rate of ventilator removal, and data pooling showed that the rate was significantly higher in the HD group than the LD group (RR: 1.74, 95%CI: 1.04 to 2.90, P=0.03; Figure 7). Three trials reported the extubation failure rate, and pooled results revealed a significantly lower extubation failure rate in the HD group compared to the LD group (RR: 0.5, 95%CI: 0.35 to 0.71, P=0.0001; Figure 8).

#### 3.1.5. Frequency of Apnea and Apnea Duration

**Figure 9:**
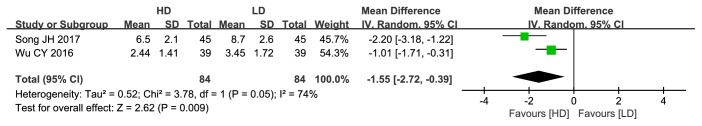
Forest plot of the frequency of apnea between HD group and LD group.

**Figure 10:**
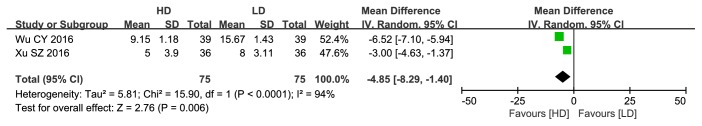
Forest plot of apnea duration between HD group and LD group.

Only 2 trials with 168 infants reported the frequency of apnea and apnea duration. The HD group demonstrated a significantly lower frequency of apnea and shorter apnea duration than the LD group (MD: -1.55, 95%CI: -2.72 to -0.39, P=0.009, Figure 9; MD: -4.85, 95%CI: -8.29 to -1.40, P=0.006; Figure 10).

### 3.2. Complications

**Figure 11:**
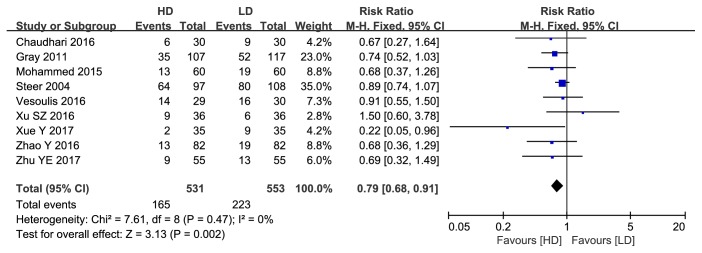
Forest plot of bronchopulmonary dysplasia between HD group and LD group.

A total of 9 articles reported the incidence of bronchopulmonary dysplasia (531 infants in the HD group and 553 in the LD group). Pooled data revealed a significantly lower incidence in the HD group (RR: 0.79, 95%CI: 0.68 to 0.91, P=0.002; Figure 11). There were no statistically significant differences in the frequencies of other complications (Table 2), such as ROP, NRC, IVH, and PVL (P>0.05).

### 3.3. Publication Bias

**Figure 12:**
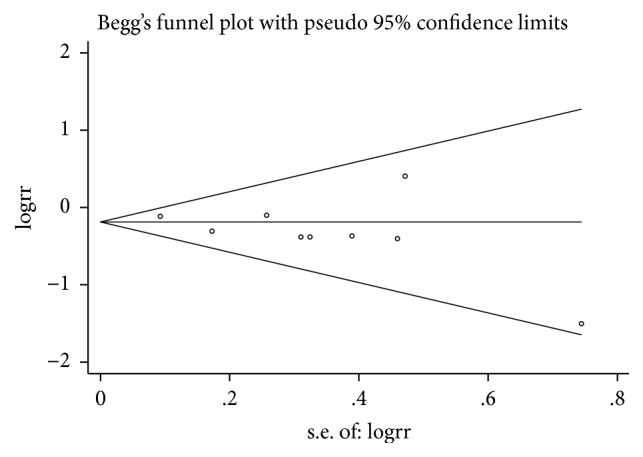
Funnel plot for publication bias test for BPD.

**Figure 13:**
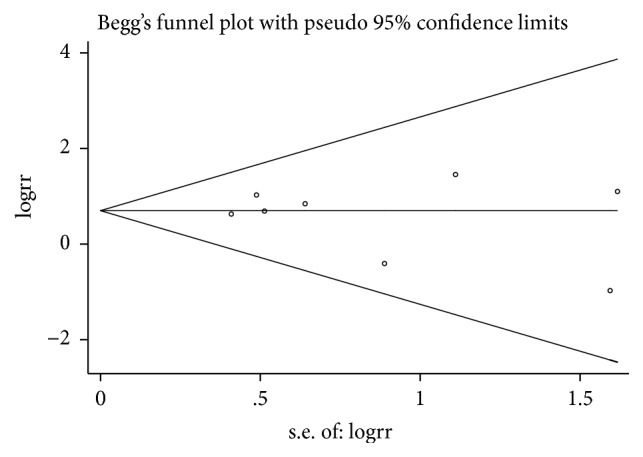
Funnel plot for publication bias test for tachycardia.

**Figure 14:**
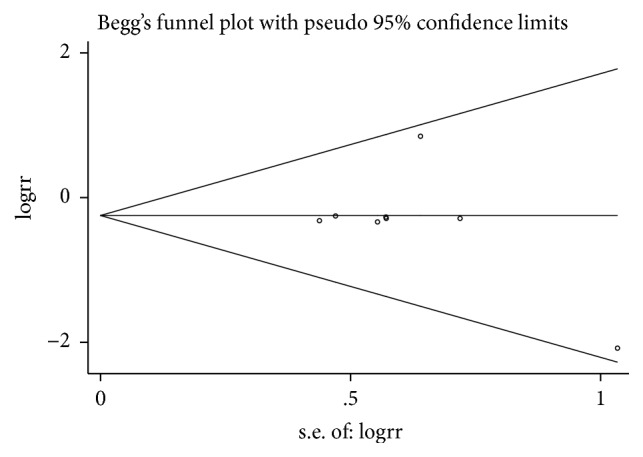
Funnel plot for publication bias test for hospital mortality.

Begg's plots are presented in Figures 12–14. Test results provided no evidence of publication bias (P_Begg's_=0.917 for BPD, Figure 12; P_Begg's_=1.000 for tachycardia, Figure 13; P_Begg's_=0.536 for hospital mortality, Figure 14).

### 3.4. Sensitivity Analysis

**Figure 15:**
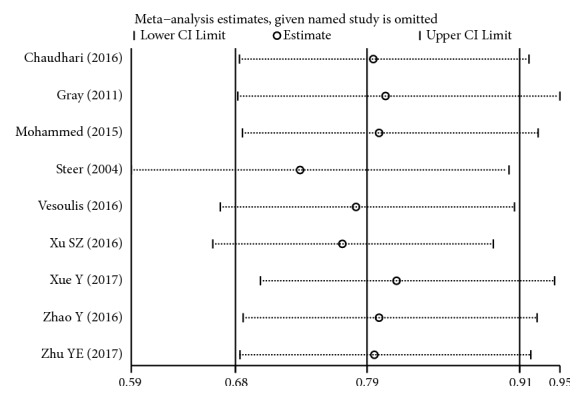
Sensitivity analysis of BPD for high versus low maintenance dose.

**Figure 16:**
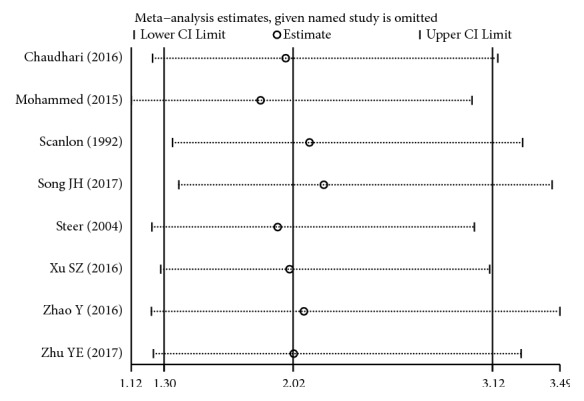
Sensitivity analysis of tachycardia for high versus low maintenance dose.

**Figure 17:**
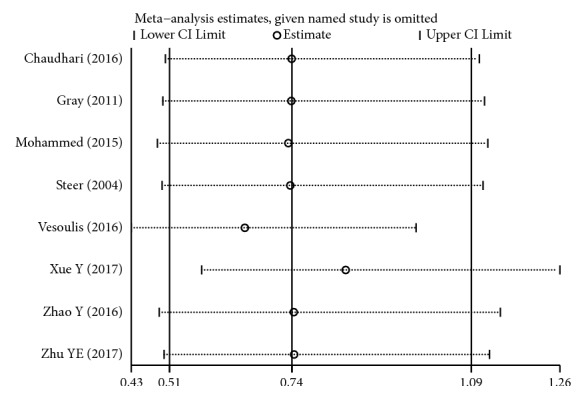
Sensitivity analysis of hospital mortality for high versus low maintenance dose.

Sensitivity analysis indicated that our current data were relatively steady and credible (Figures 15–17).

## 4. Discussion

At present, caffeine is the first choice for AOP treatment [8]. However, the maintenance dose has not been standardized [29]. Therefore, several [16–18, 20, 23] studies have examined the efficacy of different doses of caffeine for maintenance therapy of premature infants. Charles et al. reported a substantially lower extubation failure rate in a high maintenance dose group compared to a low maintenance dose group (17% versus 49%, P<0.05) as well as significantly reduced average mechanical ventilation time in the high-dose group (P<0.01) [30]. However, Mohammed et al. found that a high maintenance dose of caffeine citrate was more likely to cause adverse reactions such as tachycardia [20]. Thus, although a higher maintenance dose can improve the clinical efficiency against AOP, it may also increase the frequency of adverse reactions. At present, there is no definitive evidence from a systematic review and meta-analysis to support which maintenance dose is superior considering both efficacy and safety.

Whether the treatment is effective or not is the focus of clinicians' attention. This meta-analysis found that a higher maintenance dose of caffeine citrate (10−20 mg/kg daily) was significantly more efficacious against AOP, enhanced the success rate of ventilator removal, and decreased the extubation failure rate, apnea frequency, apnea duration, and incidence of BPD compared to a lower dose (5−10 mg/kg daily). With regard to the effective rate, sensitive analysis after excluding the outlier study also revealed a significant difference between HD group and LD group for the remaining studies with low statistical heterogeneity. Similarly, Turmen [31] found that ventilation volume per minute increased rapidly and reached a stable level in the subsequent ventilation reaction following higher maintenance doses of caffeine citrate (reaching higher blood concentrations) to premature infants. Thus, a high maintenance dose of caffeine appears more effective for promoting lung maturation in premature infants, consistent with the current results on AOP treatment.

Nonetheless, some clinicians [16–18, 20, 23] are still wary of administering large maintenance doses due to the risks of adverse reactions. Indeed, this meta-analysis found a higher incidence of tachycardia in the HD group. However, the reasons for caffeine citrate treatment are to stimulate the central nervous system, improve autonomic nerve function, promote myocardial contraction, and dilate blood vessels, which will result in increased cardiac output and blood pressure [32]. In clinical practice, children who cannot tolerate caffeine could be treated by drugs to improve tachycardia or receive a lower caffeine dose. Several trials included in this study used a maximum maintenance dose of 20 mg/kg/d, but even this dose did not necessitate discontinuation due to tachycardia, nor did it have a negative impact on the therapeutic effect and clinical outcome. The incidences of other adverse reactions, such as electrolyte disturbance, hypertension, hyperglycemia, feeding intolerance, and restlessness, also did not differ between dose groups, and there were no differences in the frequencies of severe complications, such as in-hospital mortality, ROP, NEC, IVH, and PVL. Therefore, the high maintenance dose of caffeine citrate appears safe for the treatment of AOP.

We found that Begg's Test results provided no evidence of publication bias about BPD, tachycardia, and hospital mortality. Sensitivity analysis was performed to determine the robustness of the combined data. And our meta-analysis found that the sensitivity analysis of BPD, hospital mortality, and tachycardia for high versus low maintenance dose were relatively steady and credible.

However, some limitations of this study should be acknowledged. First, the maintenance dose varied within the high- and low-dose range, which may have obscured group differences in outcomes or complication rates. Second, there were no reports on long-term outcomes such as intellectual development. Third, there were too few trials in this meta-analysis to assess some outcomes such as the success rate of removal of ventilator, extubation failure rate, frequency of apnea, apnea duration, and other rare adverse reactions. Fourth, most of the studies included are in Chinese, and the quality score is relatively low, which affects the credibility of this outcome to some extent. Therefore, more trials of high quality, multicenter, and large sample size will be included in the future. Last, we failed to assess the heterogeneity of infants regarding neonatal weight, gender, gestational age, or other factors between studies. To compensate for this deficiency, we will assess the heterogeneity of these factors in our next meta-analysis.

This meta-analysis showed that a high maintenance dose of caffeine citrate (10−20 mg/kg daily) is more effective than and at least as safe as a lower maintenance dose (5−10 mg/kg daily) for the treatment of AOP. However, owing to the limited quantity and quality of available RTCs, further study is needed to confirm these findings.

## Figures and Tables

**Table 1:** Summary of study and patient characteristics.

Study	Country	Year	Type	No. (HD/LD)	Maintenance dose (mg/kg/d)	Outcome
Zhu YE [24]	China	2017	RCT	55/55	15 vs 5	(2)(4)(5)(6)(7)(9)
Zhang LP [25]	China	2017	RCT	50/50	20 vs 10	(4)
Xue Y [26]	China	2017	RCT	35/35	15 vs 5	(2)(4)(5)(6)(7)(8)(9)
Song JH [27]	China	2017	RCT	45/45	15 vs 5	(2)(6)
Xu SZ [16]	China	2016	RCT	36/36	20 vs 10	(3)(6)(7)(8)
Wu CY [28]	China	2016	RCT	39/39	20 vs 5	(2)(3)(4)
Zhao Y [17]	China	2016	RCT	82/82	15 vs 5	(2)(4)(5)(6)(7)(9)
Vesoulis [18]	America	2016	RCT	37/37	20 vs 10	(6)(7)(8)(9)
Chaudhari [23]	China	2016	RCT	30/30	20 vs 10	(1)(2)(3)(6)(7)(8)(9)
Mohammed [20]	Egypt	2015	RCT	60/60	20 vs 10	(1)(2)(6)(7)(8)(9)
Gray [19]	Australia	2011	RCT	120/126	20 vs 5	(6)(7)(8)(9)
Steer [21]	Australia	2004	RCT	113/121	20 vs 5	(1)(3)(6)(7)(8)(9)
Scanlon [22]	Britain	1992	RCT	14/16	12 vs 6	(2)(4)(6)

Note: HD: high maintenance dose, LD: low maintenance dose; (1) the extubation failure rate, (2) the frequency of apnea, (3) apnea duration, (4) the effective rate, (5) the success rate of removal of ventilator, (6) adverse effects, (7) bronchopulmonary dysplasia, (8) complications, and (9) hospital mortality.

**Table 2:** Other complications.

	No. of trials	HD		LD		I^2^	Effect-Model	Outcomes	P
		case	total	case	total				
ROP	6	42	340	61	361	0%	Fixed	0.74[0.52,1.05]	0.09
NEC	4	10	239	19	247	0%	Fixed	0.54[0.26,1.12]	0.10
IVH	7	86	422	92	438	0%	Fixed	0.98[0.76,1.27]	0.89
PVL	3	12	119	9	120	0%	Fixed	1.35[0.59,3.07]	0.47
